# A Comparative Study on the Characterization of Nanofibers with Cellulose I, I/II, and II Polymorphs from Wood

**DOI:** 10.3390/polym11010153

**Published:** 2019-01-17

**Authors:** Haiying Wang, Suiyi Li, Tiantian Wu, Xiaoxuan Wang, Xudong Cheng, Dagang Li

**Affiliations:** College of Materials Science and Engineering, Nanjing Forestry University, Nanjing 210037, China; njfulsy@163.com (S.L.); a1030587300@163.com (T.W.); gansensama@163.com (X.W.); c347071952@163.com (X.C.)

**Keywords:** cellulose nanofibers, polymorph, cellulose II, mechanical performance, thermal property

## Abstract

Polymorphic changes in cellulose nanofibers (CNFs) are closely related to their properties and applications, and it is of interest to investigate how polymorphic changes influence their properties. A comparative study on the properties of CNFs with cellulose I, I/II, and II polymorphs from wood was conducted herein. CNFs were obtained by chemical extraction combined with a simple and efficient mechanical treatment (one pass through a grinder). This process resulted in a relatively high yield of 80–85% after a simple grinding treatment. The polymorphic changes in the CNFs and the chemical composition, morphology, tensile performances, and thermal properties were systematically characterized and compared. The X-ray diffraction and FTIR analyses verified the existence of three types of purified pulps and CNFs with cellulose I, cellulose I/II, and cellulose II polymorphs (CNF-I, CNF-I/II, CNF-II). Morphological observations presented that these three types of CNFs all exhibited high aspect ratios and entangled structures. Tensile testing showed that the CNF films all exhibited high tensile strengths, and the fracture strains of the CNF-I/II (11.8%) and CNF-II (13.0%) films were noticeably increased compared to those of the CNF-I film (6.0%). If CNF-II is used as reinforcing material, its larger fracture strain can improve the mechanical performance of the CNF composites, such as fracture toughness and impact strength. In addition, CNF-I, CNF-I/II, and CNF-II films showed very low thermal expansion in the range 20–150 °C, with the coefficient of thermal expansion values of 9.4, 17.1, and 17.3 ppm/K, respectively. Thermogravimetric analysis (TGA) revealed that the degradation temperature of CNF-I and CNF-II was greater than that of CNF-I/II, which was likely due to increased α-cellulose content. This comparative study of the characterization of CNF-I, CNF-I/II, and CNF-II provides a theoretical basis for the application of CNFs with different polymorphs and could broaden the applications of CNFs.

## 1. Introduction

Cellulose, a linear chain of ringed glucose molecules, is the most abundant natural biopolymeric material on earth [1]. Based on the different orientation of cellulose chains and hydrogen-bond networks, several polymorphs of crystalline cellulose (I, II, III, and IV) have been identified [2,3]. Cellulose I is referred to as native cellulose, which has two forms, including I_α_ and I_β_ [2]. Cellulose II is usually prepared from cellulose I through mercerization (alkali treatment) or regeneration (solubilization and recrystallization) [4]. During the mercerization process, the polymorphic transformation from cellulose I (parallel-chain structure) to cellulose II (antiparallel-chain structure) occurs via sodium hydroxide treatment [5]. Due to the different supermolecular structures of cellulose I and cellulose II, the mechanical, chemical, and thermal properties of these two types of fibers change significantly [6]. Interest in cellulose II fibers has increased owing to their excellent properties, and the application of cellulose in the chemicals industry has been further expanded [7]. Moreover, it was said that cellulose II is a better feedstock than cellulose I in biofuel production [8].

The development of nanostructured celluloses, or nanocellulose, has drawn significant interest in recent years due to its rich sources, nanoscale dimensions, high strength, light weight, renewability, and assembly performance [7,9]. There are several methods for preparing nanocellulose, including acid hydrolysis, enzymatic hydrolysis, mechanical treatment, and so on [1,10]; among these methods, mechanical treatment includes ultrasonication, high-pressure homogenization, grinding, and cryocrushing [11]. Nanocellulose is generally divided into two groups [12]: (a) cellulose nanofibers (CNFs), which are mainly obtained by mechanical fibrillation; and (b) cellulose nanocrystals (CNCs), which are mainly produced by acid treatment. CNFs consist of nanofibrils with long lengths and high aspect ratios, while CNCs consist of nanofibrils with relatively short lengths and low aspect ratios [2]. In some studies, fibrillated CNFs or CNCs with cellulose I polymorph have been treated with strong sodium hydroxide, and it was found that CNFs [13] or CNCs [3] are easily aggregated during mercerization when converting to cellulose II polymorph, and they cannot be effectively re-dispersed. CNCs with cellulose II polymorph (CNC-II) have been obtained by treating mercerized fibers using sulfuric acid hydrolysis and then high-pressure homogenization [14]. Gong et al. carried out a comparative study on the isolation and characterization of CNCs with different polymorphs [15]. Abe et al. [16] obtained CNFs with cellulose I polymorph (CNF-I) from wood using a very simple mechanical grinding treatment. However, the extraction of CNFs with cellulose II polymorph (CNF-II) is complex and requires great energy consumption [17,18], which limits their application. 

Most published studies have focused on the investigation of CNFs with cellulose I polymorph. For a more comprehensive understanding and application of fibrillated CNFs, it is necessary to study the properties of CNFs with different polymorphs. In our previous study, we reported an efficient method for extracting CNFs with cellulose II polymorph by chemical extraction combined with a simple mechanical grinding treatment. We found that the delignification plays an important role in the polymorphic transformation from cellulose I to cellulose II during mercerization and in the subsequent fibrillation of CNF-II [19]. We have described the preparation method of individual nanofibers with cellulose II polymorph, but the properties of these cellulose II nanofibers are not known. It is of interest to investigate how polymorphic changes influence their properties. Few investigations have dealt with a comparative study on the characterization of CNFs with different polymorphs. 

In this study, three types of CNFs with cellulose I, I/II, and II polymorphs were prepared using a powerful yet simple method based on our previous work. The main purpose of this study was to compare the properties of CNFs with different polymorphs. This comparative characterization would help to elucidate the chemical, physical, and morphological evolution of the properties of these three types of CNFs as they undergo fibrillation to nanofibers, and will help to broaden the potential applications of CNFs. We systematically studied these three types of CNFs, including a comparison of their chemical compositions, crystal structures, morphology, tensile performances, thermal properties, and optical properties.

## 2. Materials and Methods

### 2.1. Materials and Reagents

Wood powder from *Pinus sylvestris* var. *mongolica* Litv. was used and was ground into 80 meshes. The extracted wood powder was obtained using benzene and ethanol extraction, and was used as the raw material for all of the experiments. Benzene, ethanol, sodium chlorite (NaClO_2_, 80%), acetic acid, potassium hydroxide (KOH), and sodium hydroxide (NaOH) were of analytical grade and were used without further purification.

### 2.2. Preparation of Cellulose Nanofibers with Different Polymorphs

**Figure 1:**
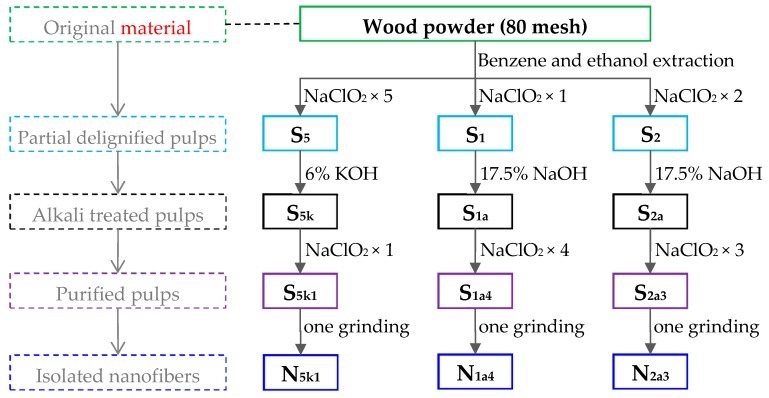
The procedure for the preparation of three types of cellulose nanofibers (CNFs) with different chemical treatments.

The isolation of purified pulps and CNFs with different polymorphs was performed as follows. The samples were divided into 3 groups and were prepared by a combination of chemical purification and mechanical treatment (one grinding treatment) according to the flowchart shown in Figure 1. During the whole process, the samples were filtered and rinsed with distilled water until the residues were neutral. In this study, the NaClO_2_/alkali/NaClO_2_-treated samples were designated as Sxyz, where x = 5, 1, or 2 (the number of acidified NaClO_2_ pretreatments), y = k = 6 wt % KOH treatment (or y = a = 17.5 wt % NaOH), and z = 1, 4, or 3 (the number of further NaClO_2_ treatments). Moreover, the samples that were further fibrillated by a single grinding treatment were designated as Nxyz. For instance, a sample receiving 5 NaClO_2_/KOH/1 NaClO_2_ treatments was designated as S_5k1_. Furthermore, sample S_5k1_, which was further fibrillated by a single grinding treatment, was designated as N_5k1_.

Group 1 (S_5k1_): Group 1 was used as a control group. The subsequent procedure followed a method described in previous studies [16]. In brief, under acidic conditions (pH 4–5), delignification was carried out with 1 wt % NaClO_2_ solution at 75 °C for 1 h, and the process was repeated 5 times. Then, the sample was treated overnight in 6 wt % KOH at room temperature, and then treated at 80 °C for 2 h to leach hemicelluloses. Finally, the sample was further treated with 1 wt % acidified NaClO_2_ solution at 75 °C for 1 h to obtain highly purified cellulose pulps.

Group 2 (S_1a4_): Initially, under acidic conditions (pH 4–5), a portion of lignin in the wood powders was leached by 1 wt % NaClO_2_ at 75 °C for 1 h. Then, the partially delignified wood powders were soaked in 17.5 wt % NaOH at 25 °C for 12 h. Finally, the residual lignin in the sample was further removed by 1 wt % acidified NaClO_2_ at 75 °C for 1 h, and this process was repeated 4 times.

Group 3 (S_2a3_): The preparation of S_2a3_ was conducted as follows, referring to our previous studies [19]. Briefly, a portion of lignin in the wood powders was first leached by 1 wt % acidified NaClO_2_ at 75 °C for 1 h, and the process was repeated 2 times. Then, the samples were soaked in 17.5 wt % NaOH at 25 °C for 12 h. Finally, the residual lignin in the sample was further removed by 1 wt % acidified NaClO_2_ at 75 °C for 1 h, and this process was repeated 3 times.

Finally, these three groups of purified pulps were treated by the same mechanical fibrillation method, and the steps were taken as follows: an aqueous suspension with 0.8 wt % undried purified pulps was prepared and passed once through a grinder (MKCA6-3; Masuko Sangyo Co., Saitama, Japan) at 1500 rpm to obtain the CNFs [16].

### 2.3. Preparation of CNF Films

The CNF suspensions were diluted to 0.2 wt % in distilled water to fabricate films. Filter membranes with pore sizes of 0.2 μm were used. After the suspension was slowly vacuum filtered and dewatered with a glass filter, a wet CNF sheet was obtained. The wet sheet was placed between two smooth wire meshes (inner layers), and then sandwiched between two filter papers (outer layers). Finally, the films were dried at 60 °C in an oven for 48 h, while a pressure of approximately 15 kPa was applied [9]. The dry CNF films were approximately 40 μm thick and 1.48 g/cm^3^ in density. 

### 2.4. Characterization

#### 2.4.1. Chemical Composition Measurement

The chemical composition including lignin content, α-cellulose content, and hemicelluloses content of the samples at different stages was determined using the methods of our previous study [18]. At least five specimens of each material were tested, and the average values were calculated.

#### 2.4.2. X-ray Diffraction (XRD)

XRD measurement was carried out using a Rigaku X-ray diffractometer (SmartLab; Rigaku Corp., Tokyo, Japan) with CuKα radiation at 40 kV and 40 mA over the range of 2*θ* = 5–40° at a scan rate of 5°/min. The samples prepared for XRD were freeze-dried and then pressed into sheets. 

#### 2.4.3. Fourier Transform Infrared (FTIR) Spectroscopy

FTIR spectra of the samples were recorded on an FTIR instrument (Nicolet IS10, Thermo Scientific, Waltham, Massachusetts, USA) with an attenuated total reflectance (ATR) mode. Each sample was tested through an accumulation of 64 scans, with a resolution of 2 cm^−1^ at 700–4000 cm^−1^.

#### 2.4.4. Field Emission Scanning Electron Microscopy (FE-SEM)

The obtained pulps were observed using a field emission scanning electron microscope (JSM-6700F; JEOL Ltd., Tokyo, Japan). In order to prevent structural collapse during the dehydration, all of the samples were solvent-exchanged and then freeze-dried. 

#### 2.4.5. Transmission Electron Microscopy (TEM)

A drop of diluted CNF suspension (0.04–0.05 wt %) was deposited on a 200-mesh ultrathin carbon-coated copper grid. A piece of filter paper was used to absorb the excess liquid on the grid. After the sample was dried, it was imaged with a transmission electron microscope (JEM-1400, JEOL Ltd., Tokyo, Japan) operated at an accelerating voltage of 80 kV. Diameters of CNFs were calculated from TEM images using ImageJ software (National Institutes of Health, Bethesda, Maryland, USA) [20]. For each sample, 200 nanofibers were selected randomly and measured from several TEM images.

#### 2.4.6. Tensile Performance

The tensile test was conducted at a crosshead speed of 1 mm/min using a universal material testing machine (Shenzhen SANS Tensile Machine Co. Ltd., Shenzhen, China). The samples (CNF films) were 30 mm long, 6 mm wide, and 40–50 μm, and were measured with a gauge length of 20 mm. At least five CNF films of each material were tested, and the average values were calculated.

#### 2.4.7. Thermomechanical Analysis (TMA)

The coefficient of thermal expansion (CTE) of the CNF films was measured by a thermomechanical analyzer (TMA/SS6100, SII Nanotechnology Inc., Tokyo, Japan). The samples were 20 mm long and 3 mm wide with a span of 15 mm. The measurement was performed three times with a heating rate of 5 °C min^−1^ under a load of 3 g in the tensile mode in a nitrogen atmosphere. The CTE value was determined at 20–150 °C in the second run. At least five specimens of each material were tested, and the average values were calculated.

#### 2.4.8. Thermogravimetric Analysis (TGA)

The thermal stability of the CNF films was analyzed with a thermogravimetric analyzer (STA 449F3, NETZSCH Inc., Bavaria, Germany). It was measured in a nitrogen atmosphere at temperatures ranging from 30 to 500 °C with a heating rate of 10 °C/min. The weight-loss rate was obtained from the derivative of the thermogravimetric (DTG) data.

#### 2.4.9. Light Transmittance

The regular light transmittances of the film samples were measured at wavelengths from 200 to 800 nm by an ultraviolet (UV)-visible spectrometer with an integrating sphere 60 mm in diameter (U-4100; Hitachi High Tech. Corp., Tokyo, Japan). Regular transmittance was measured by placing the samples 22 cm from the entrance ort of the integrating sphere. At least five CNF films of each material were tested, and the average values were calculated.

## 3. Results and Discussion

### 3.1. Chemical Composition Measurement

A wood cell wall consists of elastic microfibrils composed of crystalline and paracrystalline cellulose and a matrix composed of hemicelluloses and lignin [21,22]. The changes in the chemical composition due to acidified NaClO_2_ and alkali treatment were evaluated. Table 1 shows the chemical composition of the samples at different stages. The extracted wood fiber (after benzene and ethanol extraction) had a greater content of lignin (25.5%) and hemicelluloses (28.1%), and a lower content of α-cellulose (45.9%) compared to those of the purified samples. With increasing numbers of NaClO_2_ treatments, the lignin content in the wood fibers gradually decreased. The lignin contents in S_1_, S_2_, and S_5_ were 17.8%, 13.0%, and 4.7%, respectively. S_2_, which received two NaClO_2_ treatments, had a lignin content of 13.0%, indicating that about half of the lignin was removed from the extracted wood. When the chemical treatments were completed, the lignin content of S_5k1_, S_1a4_, and S_2a3_ decreased to 1.2%, 3.1%, and 2.7%, respectively.

Furthermore, after extraction with 17.5 wt % NaOH solution, the α-cellulose content of the obtained S_5k1_, S_1a4_, and S_2a3_ appreciably increased to approximately 84.5%, 76.3%, and 83.6%, respectively, compared to the content before alkali treatment. This may have been attributed to the alkali treatment that led to the removal of a large amount of hemicelluloses. In addition, the remaining hemicellulose percentages of S_5k1_, S_1a4_, and S_2a3_ were 11.8%, 16.7%, and 10.9%, respectively. S_1a4_ presented a greater percentage of hemicelluloses, which may be due to the higher content of lignin that remained in S_1_, preventing the extraction of hemicelluloses during the mercerization process. The strong interaction between lignin and hemicelluloses often prevents the extraction of hemicelluloses [23].

### 3.2. X-ray Diffraction (XRD) Studies

**Figure 2:**
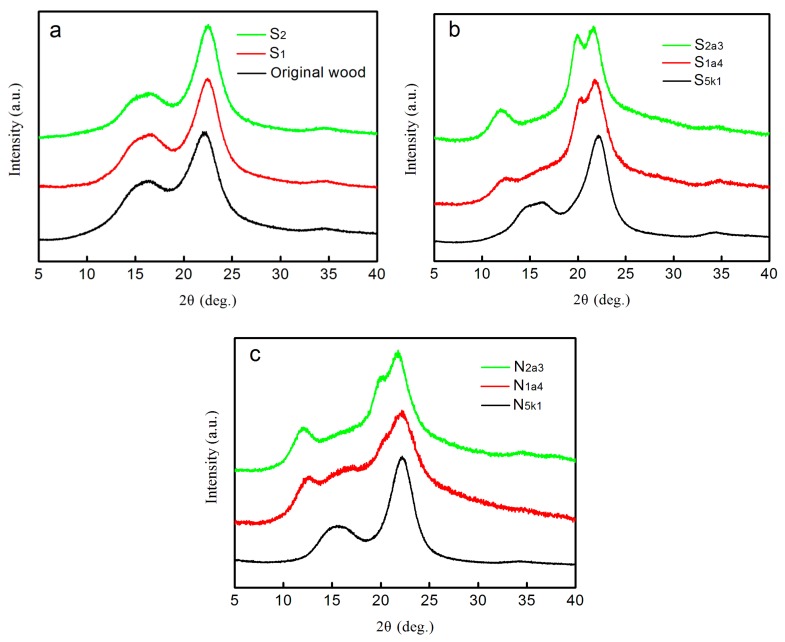
XRD patterns of the samples: (**a**) wood fibers of the original wood, S_1_, S_2_; (**b**) purified pulps, S_5k1_, S_1a4_, and S_2a3_; and (**c**) CNFs, N_5k1_, N_1a4_, and N_2a3._

XRD studies on wood fibers, purified pulps, and CNFs were conducted to study the crystalline behavior of the samples. The original wood was used as a control sample. In Figure 2a, the original wood showed the characteristic profile of cellulose I, with peaks positioned at 2*θ* = 16.01° and 22.25°, which are typical signatures of the cellulose I crystal structure [24]. Figure 2a also shows that the crystalline structures of S_1_ and S_2_ did not change during the delignification process. In Figure 2b, purified pulps with different crystalline structures were obtained after alkali treatment. After 6 wt % KOH treatment, the pulp (S_5k1_) still showed typical cellulose I characteristics. 

For S_1a4_, after 17.5 wt % NaOH treatment, the two diffraction peaks at 2*θ* = 16.01° and 22.25° decreased, and peaks at 2*θ* = 12.25° and 21.8° formed, indicating the presence of a mixture of cellulose I and II. A possible reason for the presence of a cellulose hybrid is that the lignocellulose (S_1a_) cannot be completely transferred into the swollen state, and only a portion of parallel cellulose microfibrils can rearrange to cellulose II [6]. According to the measurement of lignin content for S_1_, which received one NaClO_2_ treatment, about one-third of the lignin was removed. Thus, the swelling of cellulose in S_1_ was partially restricted by the presence of the remaining two-thirds of the lignin during the mercerizing process. For S_2_, which received two NaClO_2_ treatments, the lignin content decreased to 13.3%, showing that about half of the lignin was removed. For S_2a3_, after two NaClO_2_/NaOH/3 NaClO_2_ treatments, the sample showed diffraction peaks at 2*θ* = 12.25° (1–10), 20.2° (110), and 21.8° (200), which are speculated to represent the typical cellulose II pattern [24]. This implied that the delignified wood fibers with about half of the lignin removed (S_2_) were transformed into the cellulose II form successfully after 17.5 wt % NaOH treatment [19].

In Figure 2c, after one grinding treatment, the XRD patterns of the obtained N_5k1_, N_1a4_, and N_2a3_ were fairly similar to those of the purified pulps (S_5k1_, S_1a4_, and S_2a3_). This indicated that the mechanical fibrillation caused little damage to the crystalline structure of the CNFs, which is consistent with the previous studies [25]. Therefore, CNFs with cellulose I, cellulose I/II, and cellulose II polymorphs were obtained. In this study, CNFs with cellulose I, I/II, and II polymorphs were designated as CNF-I, CNF-I/II, and CNF-II, respectively.

### 3.3. Fourier Transform Infrared (FTIR) Spectroscopy

**Figure 3:**
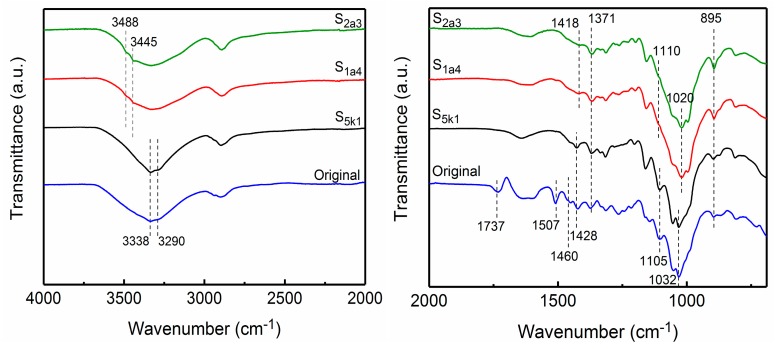
FTIR spectra of the samples: original wood, S_5k1_, S_1a4_, and S_2a3_.

FTIR spectroscopy was used to analyze the changes in the chemical constituents and to verify the crystalline structure of the samples by different treatments. Figure 3 shows comparisons of a spectra (within the range 4000–2000 cm^−1^ and 2000–700 cm^−1^) of the original wood, S_5k1_, S_1a4_, and S_2a3_. The peaks at 1507 and 1460 cm^−1^ in the spectrum of the original wood corresponded to the aromatic ring vibration and –CH_2_ deformation vibration of the lignin [25,26,27,28]. The disappearance of the peak at 1507 cm^−1^ in purified pulps (S_5k1_, S_1a4_, and S_2a3_) indicated that the lignin was almost removed after a series of chemical treatments. The peak at 1737 cm^−1^ relates to either the acetyl and uronic ester groups of the hemicellulose or to the ester linkage of the carboxylic groups of the ferulic and p-coumaric acids of lignin and/or hemicellulose [25,27,28]. The absence of the peak at 1737 cm^−1^ is attributed to the leaching of hemicelluloses after alkali treatment [25,26]. The dominant peaks of –CH and –C_1_H deformation vibration of cellulose at 1371 and 895 cm^−1^ were seen in the entire spectra [6].

After alkali treatment, characteristics in crystal structure transition from cellulose I to cellulose II were observed within 3600–3000 cm^−1^, which was attributed to hydrogen-bond stretching [6]. For the original wood and S_5k1_, the peaks corresponding to the intermolecular hydrogen bonds of 6-OH···O-3′ and the intramolecular hydroxyl groups of 3-OH…O-5 were seen at 3293 and 3338 cm^−1^ [6,29], showing the characteristics of cellulose I. When 17.5 wt % NaOH was adopted, for S_2a3_ and S_1a4_, the band at 3338 cm^−1^ disappeared, and new absorbance bands at 3445 and 3488 cm^−1^ related to the vibration of 2-OH···O-6 intramolecular hydrogen bond appeared, indicating the characteristics of cellulose II [6,30]. Moreover, the bands at 1105 cm^−1^ related to anti-symmetric ring stretch, and 1032 cm^−1^ originating from C–O–C pyranose ring skeletal vibration shifted to 1110 and 1020 cm^−1^, respectively, implying the changes of cellulose crystal structure [6,29]. In addition, the band assigned to –CH_2_ bending with aromatic ring stretching vibrated at 1428 cm^−1^, turned into a weaker band, and shifted to 1418 cm^−1^ after crystal structure transition [6]. Furthermore, the peaks at 3445 and 3488 cm^−1^ (referring to the cellulose II polymorph) in S_2a3_ were more noticeable than those in S_1a4_. These results are consistent with the results of the XRD studies. 

**Figure 4:**
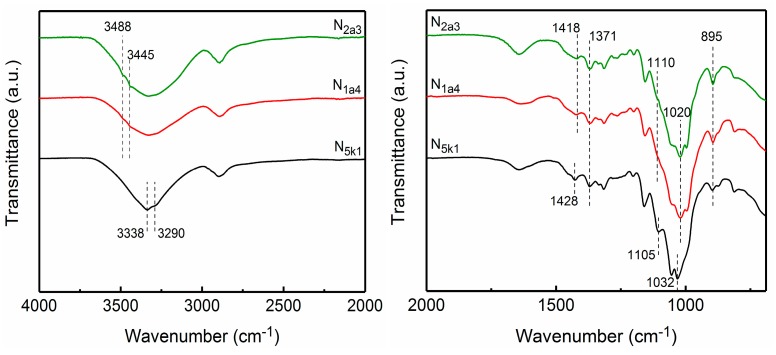
FTIR spectra of the fibrillated CNFs: N_5k1_, N_1a4_, and N_2a3_.

Figure 4 exhibits the spectra (within the range 4000–2000 cm^−1^ and 2000–700 cm^−1^) of the fibrillated CNFs, including N_5k1_, N_1a4_, and N_2a3_. After mechanical nanofibrillation by one grinding treatment, the spectra of the CNFs (N_5k1_, N_1a4_, and N_2a3_) were fairly close to those of the purified pulps (S_5k1_, S_1a4_, and S_2a3_), indicating that the molecular structures of cellulose were maintained in the case of the mechanical grinding treatment.

### 3.4. The Morphology of Pulps and Fibrillated Nanofibers

The X-ray diffraction results shown in Figure 2 indicate the presence of three types of purified pulps and CNFs with cellulose I, cellulose I/II, and cellulose II polymorphs. FE-SEM and TEM observations were used to study the morphological features of these samples.

**Figure 5:**
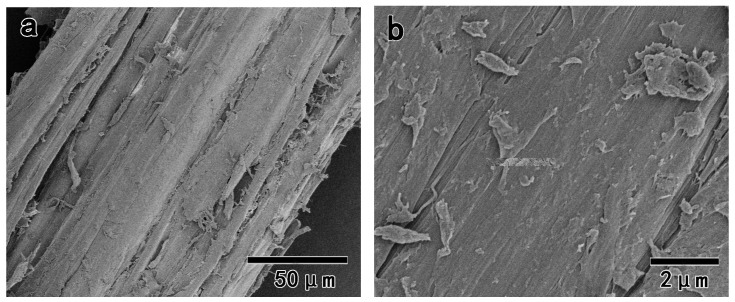
Field emission scanning electron microscopy (FE-SEM) images of the extracted wood fiber: (**a**) 600× magnification; (**b**) 10,000× magnification.

**Figure 6:**
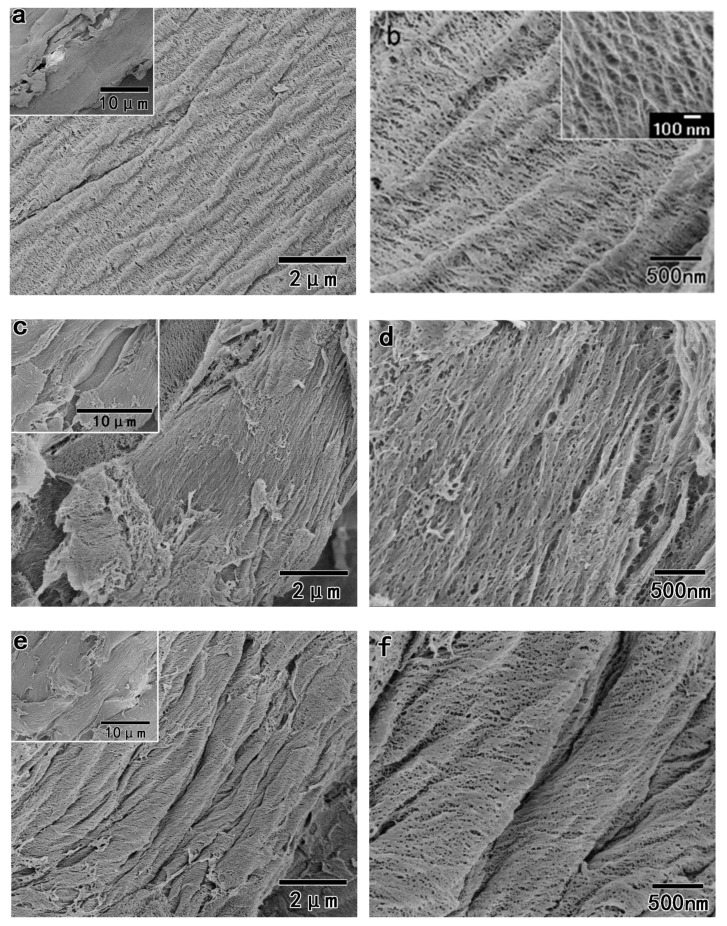
FE-SEM images of the purified pulps: (**a**,**b**) S_5k1_; (**c**,**d**) S_1a4_; and (**e**,**f**) S_2a3_.

Figure 5 shows the FE-SEM images of the wood fiber after benzene and ethanol extraction, which showed a relatively smooth surface since the extracted wood was coated by lignin and hemicelluloses. After the removal of lignin by NaClO_2_ treatment, Na^+^ ions more easily penetrated into the cellulose chains during the subsequent alkali treatment. Figure 6 shows the purified pulps (S_5k1_, S_1a4_, and S_2a3_) after NaClO_2_/alkali/NaClO_2_ treatments. For S_5k1_, cellulose microfibril bundles were clearly observed on the surface of the purified pulps. The widths of the cellulose microfibril bundles were approximately 10–30 nm, which were calculated from FE-SEM images, as shown in Figure 6b, using ImageJ software [20]. This was consistent with the previous report, which said that in cell walls, van der Waals and intermolecular hydrogen bonds promote parallel stacking of multiple cellulose chains forming elementary fibrils that further aggregate into larger microfibrils (with a diameter of 5–50 nm) [1]. In comparison, the surface of S_2a3_ become slightly aggregated, which could be attributed to the strong alkali (17.5 wt % NaOH) treatment that may cause the interdigitation of the adjacent cellulose microfibrils in the cell wall [5,31]. The aggregation of S_1a4_, as shown in Figure 6e,f, surfaces was less noticeable than that of the S_2a3_, as shown in Figure 6c,d, because of the higher content of lignin in S_1a4_, which may prevent the interdigitation of cellulose microfibrils during the alkali treatment. 

**Figure 7:**
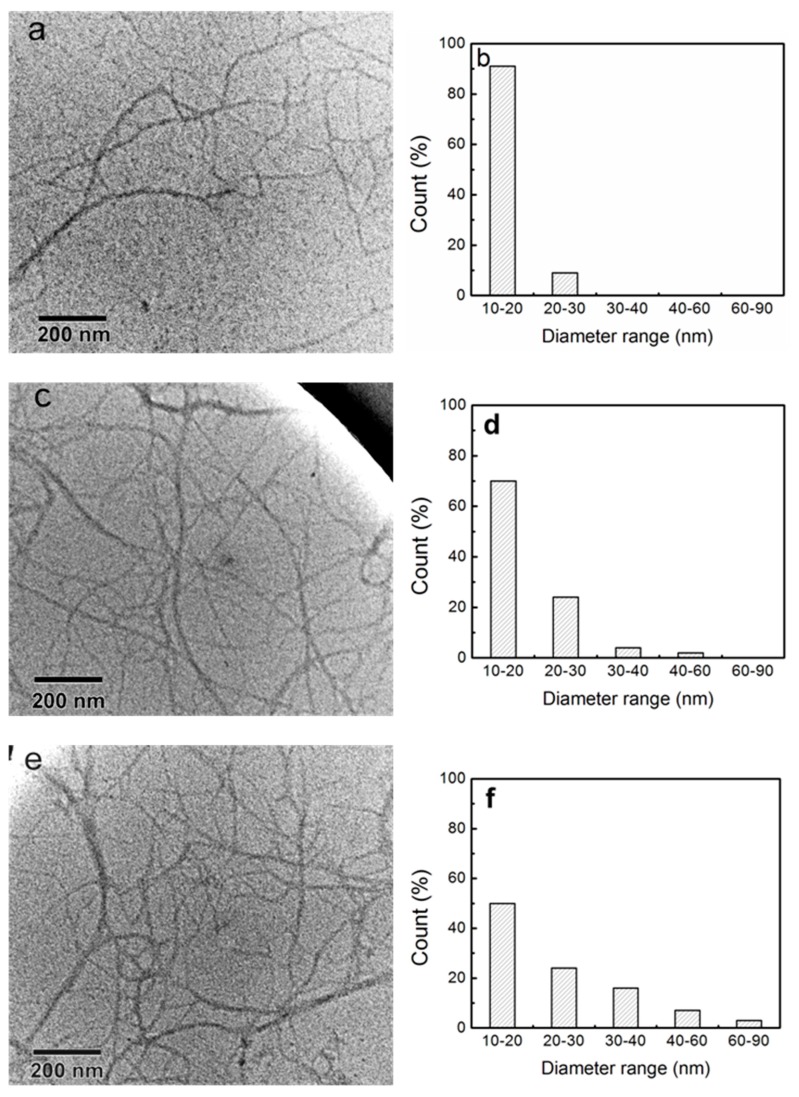
TEM images and diameter distributions of the CNFs: (**a**,**b**) CNF-I; (**c**,**d**) CNF-I/II; and (**e**,**f**) CNF-II.

Figure 7 shows TEM images and diameter distributions of the fibrillated CNFs with different polymorphs after one mechanical grinding treatment. These three types of CNFs all exhibited high aspect ratios, and the lengths of the CNFs were several microns. CNF-I (N_5k1_) showed a uniform width of 10–30 nm, as shown in Figure 7a,b. Notably, CNF-I/II (N_1a4_), with a width of 10–60 nm, was successfully obtained, as shown in Figure 7c,d. CNF-II (N_2a3_), with a width of 10–90 nm, was obtained using a simple mechanical grinding treatment, as shown in Figure 7e,f. For S_1a4_ and S_2a3_, 17.5 wt % NaOH was used to mercerize the cellulose fibers, which may cause the interdigitation of the neighboring microfibrils, resulting in a larger width of CNFs after mechanical fibrillation. According to the chemical composition measurements, the remaining hemicellulose percentages of S_1a4_ and S_2a3_ were 16.9% and 10.6%, respectively. S_1a4_ showed a greater percentage of hemicelluloses, which may facilitate the nano-fibrillation of CNFs [32].

In previous studies, cellulose II nano-particles have been produced by other methods. Yue et al. [14] hydrolyzed mercerized cotton fibers with sulfuric acid hydrolysis and then high-pressure homogenization. The resulting rod-like cellulose II nanocrystals (CNC-II) were 76 ± 20 nm in length, and 14.2 ± 3.0 nm in width. Gong et al. [15] obtained CNC-II by treating mercerized cellulose using sulfuric acid hydrolysis, and the resulting CNC-II showed a rod-like shape with short lengths of 100–250 nm and widths of 5–15 nm. In the present study, the obtained three types of CNFs with cellulose II polymorph all exhibited a length of a few microns and widths of several tens of nanometers. These CNF-II, with a high aspect ratio, may have better reinforcing properties for composites than the rod-like CNCs.

### 3.5. Tensile Performance

**Figure 8:**
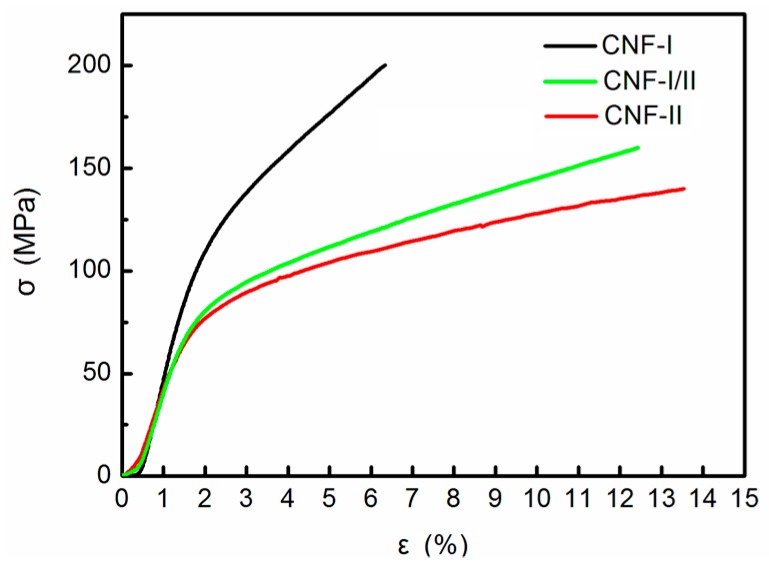
Tensile stress-strain curves of CNF-I, CNF-I/II, and CNF-II films.

In the present study, CNF films with cellulose I, I/II, and II polymorphs were obtained, then their tensile performances were examined (the film thickness was approximately 40 μm). The densities of all CNF films were about 1.48 g/cm^3^. The CNF-I film was used as a control sample. Figure 8 shows the stress-strain curves of N_5k1_, N_1a4_, and N_2a3_ films with different polymorphs. Table 2 shows the average tensile strength, Young’s modulus, and fracture strain obtained from the initial stress-strain curves. The N_5k1_ (CNF-I) film showed a tensile strength of 202 MPa, a Young’s modulus of 9.5 GPa, and a fracture strain of 6.0%. The N_1a4_ and N_2a3_ films also showed high tensile strengths (CNF-I/II: 157 MPa; and CNF-II: 137 MPa). The Young’s moduli of the CNF-I/II and CNF-II films decreased after strong NaOH treatment as compared to that of the CNF-I film. Furthermore, the superiority of the fracture strain values of the CNF-I/II and CNF-II films is shown in the stress-strain curves. The fracture strains of the CNF-I/II (11.8%) and CNF-II (13.0%) films were clearly increased compared to that of the CNF-I film (6.0%). We calculated the fracture work of CNF films according to the stress-strain curves. It was found that the energy required for the fracture of CNF-I/II and CNF-II films is much larger than that of CNF-I films, which indicates that CNF-I/II and CNF-II films have better toughness than CNF-I films.

In previous studies, the Young’s moduli of ramie and curaua fibers treated with strong alkali decreased, while the strain at fracture increased compared with untreated fibers [33,34]. Our results were consistent with these findings with values measured on the superstructures of the nanofibers. This indicated that the high toughness of the CNF-I/II and CNF-II films was attributed to the crystalline structural changes in the nanofibers. The cellulose II crystal may have become softer after strong alkali treatment, and the amorphous components increased, resulting in a high toughness [18].

These results showed that the tensile performances were affected by the crystalline structural changes in the CNF films. The increase of the fracture strain of CNF-II may improve the mechanical performances of the nanocomposites, such as impact strength and fracture toughness, if the CNF-II are used as reinforcing materials.

### 3.6. Thermomechanical Analysis (TMA)

One of the advantages of CNFs is their extremely low thermal expansion. Sun et al. [35] reported that the CTE of the CNF/CNC films from wood pulps was 11.86–17.65 ppm/K. The CTE mismatches and large CTE values among various components may lead to the malfunction of electronic devices and a decrease of the mechanical properties of composites [36]. Thus, it is necessary to study the coefficient of thermal expansion (CTE) of the CNFs if they are used as reinforcing materials in composites or sustainable electronic applications.

In this study, the thermal expansion of CNF-I, CNF-I/II, and CNF-II films was studied and compared, and the results are shown in Table 1. N_5k1_, with the cellulose I polymorph, exhibited a much smaller thermal expansion (9.4 ppm/K) between 20 and 150 °C. On the other hand, CNF-I/II (N_1a4_) and CNF-II (N_2a3_) also presented small CTE values of 17.1 and 17.3 ppm/K, respectively. The CTE values slightly increased when the crystalline structure of CNFs was converted from cellulose I to cellulose II, which may be due to the increase in the amorphous region of cellulose resulting from the strong alkali (17.5 wt % NaOH) treatment.

### 3.7. Thermogravimetric Analysis (TGA) 

**Figure 9:**
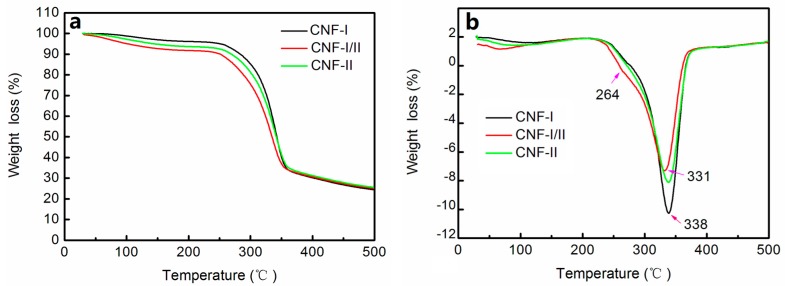
TG (**a**) and DTG (**b**) curves of CNF-I, CNF-I/II, and CNF-II films.

It is important to study the thermal properties of CNFs for evaluating their applicability in the processing of bio-composites, in which the processing temperature of thermoplastic polymers is increased to more than 200 °C [25]. Figure 9 exhibits the TG and DTG curves of the CNF-I, CNF-I/II, and CNF-II films. All TG curves presented an initial slight decrease from 30 to 150 °C, attributed to a mass loss of the absorbed moisture or the residues of low molecular weight compounds from the isolation procedures. From 150 to 500 °C, the degradation process began in the cellulose, hemicelluloses, and the associated linked water [37]. Due to the low decomposition temperature of the remaining hemicelluloses and lignin, the samples began to degrade from approximately 210 °C. As shown in Figure 9, the decomposition process was mild before 250 °C. In the main decomposition region from 250 to 500 °C, cellulose decomposed due to the breakdown of the molecular structure. In Figure 9b, the DTG curves of the CNF-I and CNF-II films showed cellulose decomposition with the maximum thermal degradation occurring at 338 °C. For the CNF-I/II film, the maximum thermal degradation occurred at 331 °C. The higher degradation temperature of CNF-I and CNF-II was likely due to the greater α-cellulose content.

Yang et al. [38] analyzed the thermal properties of hemicelluloses and showed that the decomposition peak of hemicelluloses reached a maximum mass loss rate at 268 °C. In the DTG curves in Figure 9b, the CNF-I/II film showed a clear broadening or shoulder peak at 264 °C, which may have been due to the decomposition of hemicelluloses. For CNF-I and CNF-II films, the DTG curve showed a smaller shoulder peak at approximately 264 °C, which could have been due to the lower content of hemicelluloses. This likely resulted in improved thermal properties for CNF-I and CNF-II as the decomposition of hemicellulose begins at a much lower temperature [39] than that of cellulose [39]. 

### 3.8. Light Transmittance

**Figure 10:**
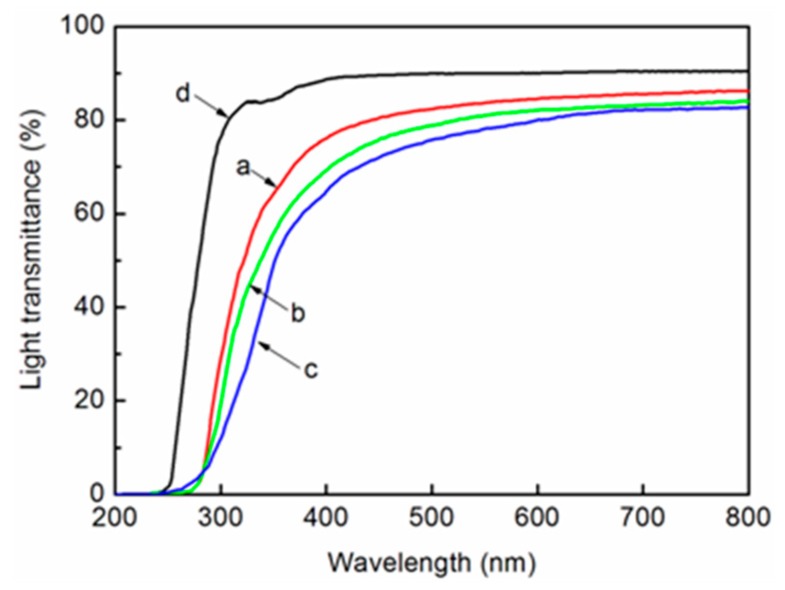
Regular light transmittances of the nanocomposites made with fibrillated (a) CNF-I, (b) CNF-I/II, (c) CNF-II, and (d) acrylic resin.

It was clarified that the light transmittances of the composites prepared with fibrillated pulp and acrylic resin show good agreement with the degree of nanofibrillation [32]. Hence, the regular light transmittance of the nanocomposite was evaluated as an indirect estimation of the fiber width distribution. We produced a nanocomposite with acrylic resin using CNF-I, CNF-I/II, and CNF-II as fillers, according to the method by Iwamoto et al. [32]. The light transmittances of the acrylic resin and the nanocomposites are shown in Figure 10.

**Figure 11:**
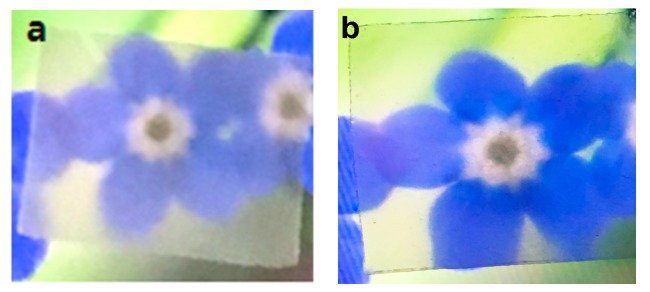
(**a**) Image of the CNF-II film; (**b**) image of the CNF-II/acrylic resin composite film.

The acrylic resin, as shown in Figure 10d, was used as a control sample and showed the highest light transmittance at a wavelength of 600 nm. The CNF-I, CNF-I/II, and CNF-II nanocomposites (thickness: 60 µm) displayed 84.7%, 82.6%, and 80.1% light transmission, respectively, at a wavelength of 600 nm, as shown in Figure 10a–c. The color of the developed film (for example: CNF-II film) was translucent white, as shown in the following image in Figure 11a. Figure 11b shows the nanocomposite made with acrylic resin using cellulose II nanofibers as a filler, which is transparent. This confirms that the CNFs obtained from wood powders in this study are uniformly nanofibrillated using the appropriate delignification technique and mechanical treatment.

## 4. Conclusions

CNFs with cellulose I, I/II, and II polymorphs were obtained by chemical purification and one mechanical grinding treatment. It resulted in a relatively high yield of approximately 80–85% after a simple grinding treatment, which showed less energy consumption compared to previous studies [17,18]. The obtained three types of CNFs have similar morphology with long lengths and a high aspect ratio. This comparative study on the characterization of CNF-I, CNF-I/II, and CNF-II provides a theoretical basis for the application of CNFs with different polymorphs. These three types of CNFs also have other advantageous properties, including light weight, high strength, fully bio-based, easily recyclable, and biodegradable properties, and are expected to be useful in the field of biomaterials, nanocomposites, barrier materials, electronic materials, super capacitors, and others. In addition, the increase of the fracture strain of CNF-II may improve the mechanical performance of the nanocomposites, such as impact strength and fracture toughness, if they are used as reinforcing materials.

## Figures and Tables

**Table 1:** Chemical composition of the samples at different stages.

Sample	α-Cellulose (%)	Lignin (%)	Hemicelluloses (%)
Extracted wood	45.9 (1.8 *^a^*)	25.5 (1.7 *^a^*)	28.1 (2.1 *^a^*)
S_1_	50.2 (1.7 *^a^*)	17.8 (1.4 *^a^*)	29.2 (1.5 *^a^*)
S_2_	52.6 (1.8 *^a^*)	13.0 (1.2 *^a^*)	30.4 (1.8 *^a^*)
S_5_	64.5 (2.1 *^a^*)	4.7 (0.8 *^a^*)	28.3 (1.9 *^a^*)
S_5k1_	84.5 (1.2 *^a^*)	1.2 (0.2 *^a^*)	11.8 (2.2 *^a^*)
S_1a4_	76.3 (2.5 *^a^*)	3.1 (0.7 *^a^*)	16.7 (2.2 *^a^*)
S_2a3_	83.6 (2.3 *^a^*)	2.7 (0.6 *^a^*)	10.9 (1.6 *^a^*)

*^a^* The standard deviation value of five samples.

**Table 2:** Average tensile properties of CNFs with different polymorphs.

Sample	Polymorph	CTE (ppm/K)	Tensile Strength (MPa)	Young’s Modulus (GPa)	Fracture Strain (%)
N_5k1_	CNF-I	9.4	202 (11 *^a^*)	9.5 (0.7 *^a^*)	6.0 (0.7 *^a^*)
N_1a4_	CNF-I/II	17.1	157 (7 *^a^*)	6.8 (0.6 *^a^*)	11.8 (1.4 *^a^*)
N_2a3_	CNF-II	17.3	137 (6 *^a^*)	6.0 (0.5 *^a^*)	13.0 (1.1 *^a^*)

*^a^* The standard deviation value of ten samples. CTE: coefficient of thermal expansion.
